# Tumor necrosis is an underappreciated histopathologic factor in the grading of chondrosarcoma

**DOI:** 10.1186/s12885-023-11022-x

**Published:** 2023-06-23

**Authors:** Alexander L. Lazarides, Bijan Abar, Bruce Leckey, John T. Martin, Evelyna G. Kliassov, Brian E. Brigman, William C. Eward, Diana M. Cardona, Julia D. Visgauss

**Affiliations:** 1 Department of Sarcoma, Moffitt Cancer Center, CSB 6th Floor, 12902 USF Magnolia Drive, Tampa, FL 33612 USA; 2 Department of Orthopaedic Surgery, Duke University Medical Center, Durham, NC USA; 3 Forefront Dermatology, Manitowoc, WI USA; 4 Department of Pathology, Duke University Medical Center, Durham, NC USA; 5 Department of Orthopaedic Surgery, Rush University Medical Center, Chicago, IL USA

**Keywords:** Chondrosarcoma, Histopathologic features, Tumor necrosis, Lymphovascular invasion

## Abstract

**Background:**

Cartilaginous neoplasms can be challenging to grade; there is a need to create an evidence-based rubric for grading. The goal of this study was to identify histopathologic features of chondrosarcoma that were associated with 5-year survival and to compare these to traditional patient, tumor and treatment variables.

**Methods:**

This was a retrospective review of all patients undergoing surgical resection of a primary chondrosarcoma with at least 2 years of follow up. All specimens were independently reviewed by two pathologists and histopathologic features scored. Univariate and multivariate analyses were performed utilizing Kaplan Meier and proportional hazards methods to identify variables associated with 5-year disease specific survival (DSS) and disease free survival (DFS).

**Results:**

We identified 51 patients with an average follow up of 49 months eligible for inclusion. 30% of tumors were low grade, 45% were intermediate grade, and 25% were high grade. In a univariate analysis considering histopathologic factors, higher tumor mitotic rate (HR 8.9, *p* < 0.001), tumor dedifferentiation (HR 7.3, *p* < 0.001), increased tumor cellularity (HR 5.8, *p* = 0.001), increased tumor atypia (HR 5.8, *p* = 0.001), LVI (HR 4.7, *p* = 0.04) and higher tumor necrosis (HR 3.7, *p* = 0.02) were all associated with worse 5-year DSS. In a multivariate analysis controlling for potentially confounding variables, higher tumor necrosis was significantly associated with disease specific survival survival (HR 3.58, *p* = 0.035); none of the factors were associated with DFS.

**Conclusions:**

This study provides an evidence-based means for considering histopathologic markers and their association with prognosis in chondrosarcoma. Our findings suggest that necrosis and LVI warrant further study.

## Background

Chondrosarcoma is a mesenchymal tumor that represents the second most common primary bone cancer. Grading of chondrosarcoma has remained largely unchanged over the past 40 years. In a landmark study by Evans et al., 71 cases of chondrosarcoma were classified on the basis of their histopathology and tumor behavior, including mitotic rate, presence of myxoid, cellularity and atypia [1]. This study found that grade appeared to correlate with prognosis and that these tumors could in fact be reliably distinguished based on their histopathologic features. However, these features were assigned arbitrarily, without any statistical evidence to support their use and without a rubric to reliably recapitulate this grading scheme. Another major limitation of the study was that it didn’t clearly investigate the contribution of individual histopathologic markers to prognosis, even neglecting to consider features such as necrosis or lymphovascular invasion (LVI) in the consideration of “grade”. These features have been demonstrated to be of particular importance in a range of other bone and soft tissue sarcomas, with both tumor necrosis and lymphovascular invasion being associated with significantly poorer survival [2–7].

Adding to the inherent challenges of grading chondrosarcomas, dedifferentiated chondrosarcomas have been debated in the literature. First, there is inherent variability in grading nomenclature of dedifferentiated tumors. Typical grading of sarcomas is reported as grades 1, 2, and 3; or low, medium, and high respectively. In the extremities, low grade tumors are now considered as distinct entitities known as atypical cartilaginous tumors. Some institutions respect this grading scheme and assign dedifferentiated tumors a grade of 3. However, others have adopted the use of grade 4 for dedifferentiated tumors, delineating a higher level of aggressiveness from other central conventional chondrosarcomas [8]. Additionally, there is debate as to whether dedifferentiated chondrosarcoma is a progression of central conventional chondrosarcoma, or a separate entity altogether [9–11]. Taken together, this adds to the inherent diagnostic uncertainty of chondrosarcomas and the difficulty with prognostication.

As a result, the general consensus is that cartilaginous neoplasms are particularly challenging to grade [12]. In one study by the Skeletal Lesions Interobserver Correlation among Expert Diagnosticians Study Group, nine musculoskeletal pathologists demonstrated a “weak” inter rater reliability when grading cartilaginous neoplasms both with respect to distinguishing benign vs. malignant and high vs. low grade. These findings have been recapitulated in subsequent studies [13, 14]. Clearly, there is a need to reevaluate grading of chondrosarcomas, in particular the individual contributing histopathologic features, to create an evidence-based rubric for grading. However, such markers in chondrosarcoma are of uncertain prognostic value and the histopathologic features that are most associated with survival have not been well established.

Our goal was to evaluate histopathologic and clinical markers that may help us improve grading and staging to improve categorization and prognostication in these tumors. Specifically, the goal of this study was to identify histopathologic features of chondrosarcoma that were associated with 5-year survival and to compare these to traditional patient, tumor and treatment variables.

## Methods

This study received institutional review board approval. We retrospectively reviewed all patients undergoing surgical resection of a primary chondrosarcoma at a single tertiary care referral center from 2006–2018. Tumor locations included extremity (shoulder girdle including scapula and lateral clavicle through wrist and proximal femur through distal tibia), chest (medial clavicle, sternum and ribs) and pelvis (not including the sacrum). Patients were considered eligible for inclusion if they had at least 2 years of follow up available. Patients were excluded from analysis if they had insufficient follow up or incomplete treatment details. We excluded patients with preexisting genetic conditions (i.e. multiple hereditary exostosis, Olliers, etc.), secondary chondrosarcomas arising from osteochondromas, and non-conventional subtypes (e.g., extraskeletal myxoid chondrosarcoma, clear cell, and mesenchymal). Patients were also excluded for whom surgical specimens were not available for histopathologic review.

A histopathologic review was performed for each tumor specimen by two pathologists who were blinded to the patients’ outcomes. For each tumor, the following were noted: tumor grade, degree of cellularity, severity of atypia, percent of necrosis, number of mitoses, presence of LVI, presence of dedifferentiation and/or myxoid change. While low grade tumors in the extremities are now classified as atypical cartilaginous tumors, in this study, grade was considered as low, intermediate or high [1]. Differentiation between low grade chondrosarcoma and enchondromas was made based on a combination of pathologic and radiographic parameters, as has been suggested previously [15]. Cellularity and atypia were qualitatively assessed as minimal, mild, moderate, or marked. Necrosis was quantified as absent, < 10%, 10–50%, 50–90%, > 90%. Mitotic index was counted per 10 high powered fields. LVI and dedifferentiation was considered as present or absent. Myxoid composition was graded as absent, < 10%, 10–50%, 50–90%, > 90%. “Traditional” histopathologic markers were considered as mitotic index, cellularity, atypia, presence of myxoid and dedifferentiation. “Novel” histopathologic markers were considered as LVI and tumor necrosis [1].

The outcome measures of interest were overall 5-year disease specific survival (DSS) and 5-year disease free survival (DFS); both measures were considered from the time of resection. DSS is defined as survival time from the primary tumor resection to confirmed death from disease or censorship. DFS is defined as the time from the primary tumor resection that the patient was alive and had no sign of local recurrence or distant metastasis. Additional variables of interest were extracted from the charts of patients and included: age, sex, race, tumor location, tumor size, margin status, use of radiation therapy and chemotherapy.

We performed Kaplan Meier analyses to identify the factors associated with 5-year DSS and 5-year DFS in univariate measures. A sensitivity analysis was performed for all continuous variables to define a cutoff point that most closely correlated with survival and recurrence for inclusion in the multivariate model. A Cox Proportional Hazards analysis was then used to identify factors independently associated with 5-year DSS and 5-year DFS. All statistical analyses were performed with JMP Pro 15 software (SAS Institute, Inc, Cary, NC).

## Results

We identified 98 patients who underwent surgical resection of chondrosarcoma of the extremities, pelvis or chest wall from 2006 to 2019. After applying exclusion criteria, 19 patients were excluded owing to insufficient follow up or incomplete treatment data and 28 patients were excluded owing to a lack of pathologic specimens available for review. This left 51 patients with an average follow up of 49 months (range 24 to 180 months) eligible for inclusion (Table 1).

**Table 1:** Descriptive characteristics of patients eligible for inclusion

Characteristic	n (%)
**Age at diagnosis (median y)**	57
**Sex**	
Female	24 (47)
Male	27 (53)
**Race**	
Caucasian	45 (88)
Other	5 (10)
Unavailable	1 (2)
**Tumor size (median cm)**	9
**Use of Radiation Therapy**	7 (14)
**Use of Chemotherapy**	12 (24)
**Tumor location**	
Extremity	31 (61)
Pelvis	12 (24)
Chest	8 (16)
**Histologic subtypes**	
Central Conventional	39 (77)
Dediff	12 (24)
**Surgical Procedure**	
Wide resection with limb salvage	50 (98%)
Curettage	1 (2%)
**Margins**	
Negative	38 (75)
Positive	12 (24)
Indeterminate	1 (2)
**Grade**	
Low	15 (29)
Intermediate	23 (45)
High	13 (26)
**Follow Up (mo) (range)**	49 (24–320)

**Fig. 1:**
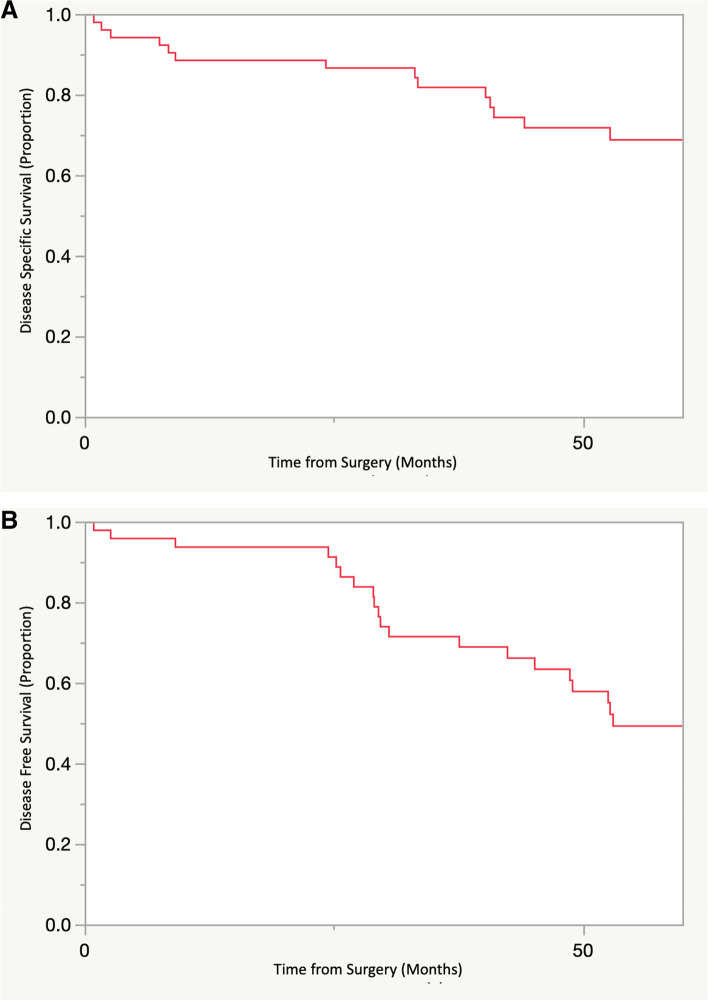
Kaplan Meier Curves demonstrating the **A**) 5-year disease specific survival and **B**) 5-year disease free survival

After independent review, 15 tumors (29%) were deemed to be low grade, 23 tumors (45%) were intermediate grade, and 13 tumors (26%) were high grade. Of the tumors that were classified as “high grade,” 12 of 13 of these tumors demonstrated the presence of a dedifferentiated component. All tumors with a dedifferentiated component were associated with a corresponding lower grade central conventional chondrosarcoma. 1 low grade chondrosarcoma was treated with curettage; the remaining cases were treated with wide resection or amputation. Margins were reported for 50 of the 51 patients (98%); of these, 12 patients (24%) had positive margins. 12 patients (24%) had a local recurrence; 14 patients (28%) developed metastatic disease. The 5-year DSS for the cohort was 69%, while the 5-year DFS for the cohort was 56% (Fig. 1A and B). The median time to metastasis was 9 months (range 1–53 months). When stratified by grade, the 5-year DSS was 92%, 71% and 40% for grades 1, 2 and 3 respectively (*p* < 0.001); the 5-year DFS was 91%, 56% and 22% for grades 1, 2, and 3 respectively (*p* < 0.001).

A descriptive review of the pathologic variables considered for the study are included in Table 2. Most tumors demonstrated low or absent necrosis (64%). The majority of tumors (83%) showed a low mitotic index. LVI was detected in only 3 patients (6%); 2 of the 3 patients with LVI died of disease within 5 years. 42% of tumors demonstrated a myxoid component.

**Table 2:** Descriptive characteristics of pathologic variables

Pathologic Factors	n (%)
Necrosis	
Absent	14 (27.4)
< 10%	19 (37.3)
10–50%	10 (19.6)
> 50%	8 (15.7)
Cellularity	
0	2 (3.9)
1	14 (27.5)
2	20 (39.2)
3	15 (29.4)
4	0
Atypia	
0	1 (1.9)
1	19 (37.3)
2	18 (35.3)
3	13 (25.5)
4	0
Mitosis	
> 5/ 10HPF	9 (17.6)
< 5/ 10HPF	42 (82.4)
LVI	
Yes	3 (5.9)
No	50 (94.1)
Myxoid Component	
Yes	20 (39.2)
No	31 (60.8)

When considering patient characteristics (Table 3), age older than 60 was associated with worse 5-year DSS (HR 3.9, *p* = 0.022) and worse 5-year DFS (HR 3.0, *p* = 0.0.22). Tumor location, race and sex were not associated with 5-year DSS or DFS. When considering tumor and treatment characteristics, tumor size > 8 cm was associated with worse 5-year DFS (HR 3.8, *p* = 0.04). When compared to low grade tumors, high tumor grade was associated with worse 5-year DSS (HR 16.1, *p* = 0.01) and DFS (HR 21, *p* = 0.004). While metastatic disease was associated with worse 5-year DSS (HR 12.9, *p* < 0.001), local recurrence was not associated with worse DSS (*p* = 0.99). Chemotherapy was only given in the setting of existing metastatic disease; when controlling for metastatic disease, it was not independently associated with 5-year DSS (*p* = 0.9). Margin status and use of radiation therapy was not associated with 5-year DSS or DFS.

**Table 3:** Univariate analysis of association of patient and treatment characteristics with 5-year disease specific survival (DSS) and disease free survival (DFS)

**Patient Parameter**	**5-y DSS (HR)**	***p***	**Lower 95%**	**Upper 95%**	**5-y DFS (HR)**	***p***	**Lower 95%**	**Upper 95%**
**Age**								
Age < 60	ref				ref			
Age > 60	3.99	**0.022**	1.23	12.97	2.84	**0.035**	1.08	7.50
**Gender**								
Male	ref				ref			
Female	0.47	0.21	0.144	1.53	0.45	0.13	0.16	1.27
**Race**								
Other	ref				ref			
Caucasian	1	0.99			0.82	0.79	0.18	3.61
**Site**								
Extremity	ref				ref			
Pelvis	1.43	0.53	0.47	4.38	1.49	0.44	0.54	4.1
Chest Wall	1	0.997			0.41	0.4	0.053	3.24
**Tumor/ Treatment Parameter**	**5-y DSS (HR)**	***p***	**Lower 95%**	**Upper 95%**	**5-y RFS (HR)**	***p***	**Lower 95%**	**Upper 95%**
**Tumor size**								
< 8	ref				ref			
> 8	1.96	0.29	0.53	7.24	3.69	**0.042**	1.05	12.99
**Grade**								
Low	ref				ref			
Intermediate	4.9	0.15	0.57	41.93	1.61	0.52	0.38	6.74
High	24.21	**0.003**	2.91	201.07	10.49	**0.002**	2.0	21.2
**Margins**								
Negative	ref				ref			
Positive	1.52	0.49	0.47	4.96	1.55	0.41	0.54	4.41
**Radiation Therapy**								
Yes	ref				ref			
No	2.37	0.35	0.31	18.24	00.52	0.30	0.163405	1.93615
**Chemotherapy**								
Yes	ref				ref			
No	0.32	**0.043**	0.11	0.96	0.19	** < 0.001**	0.07	0.49
**Local Recurrence**								
No	ref				-	-	-	-
Yes	1	0.99			-	-	-	-
**Distant Metastasis**								
No	ref				-	-	-	-
Yes	13.69	** < 0.001**	3.03	61.86	-	-	-	-

When compared to the initial histopathologic report, the grade changed for 5 patients (9%) upon re-review by our pathologists; all patients were upgraded to a higher grade on rereview. In a univariate analysis considering histopathologic factors (Table 4), higher tumor mitotic rate (HR 8.9, *p* < 0.001), tumor dedifferentiation (HR 7.3, *p* < 0.001), increased tumor cellularity (HR 5.8, *p* = 0.001), increased tumor atypia (HR 5.8, *p* = 0.001), LVI (HR 4.7, *p* = 0.04) and higher tumor necrosis (HR 3.7, *p* = 0.02) were all associated with worse 5-year DSS. Tumor necrosis and LVI were not associated with 5-year DFS, while cellularity (HR 5.64 *p* < 0.001), atypia (HR 5.75, *p* < 0.001), mitotic index (HR 10.04, *p* < 0.001) and dedifferentiation (HR 6.87, *p* < 0.001) correlated with DFS. Chondrosarcoma with a myxoid component had no association to either DSS or DFS.

**Table 4:** Univariate analysis of association of pathologic characteristics with 5-year disease specific survival (DSS) and disease free survival (DFS)

**Pathologic Factors**	**5-y DSS (HR)**	***p***	**Lower 95%**	**Upper 95%**	**5-y DFS** (**HR)**	***p***	**Lower 95%**	**Upper 95%**
***Necrosis***								
Absent	ref				ref			
< 10%	3.05	0.32	0.34	27.28	2.37	0.29	0.48	11.74
> 10%	7.1	0.07	0.89	56.79	3.96	0.08	0.86	18.36
***Necrosis***								
Low (< 10%)	ref				ref			
High (> 10%)	3.29	**0.034**	1.07	10.08	2.24	0.10	0.87	5.84
***Cellularity***								
0 to 1	ref				ref			
2	1	0.99			1	0.99		
3	3.62	**0.03**	1.17	11.26	3.06	**0.026**	1.14	8.17
***Cellularity***								
Low (< / = 2)	ref				ref			
High (> 2)	6.36	**0.001**	2.1	19.68	5.64	** < 0.001**	2.11	15.06
***Atypia***								
0 to 1	ref				ref			
2	5.03	0.14	0.59	43.1	8.07	0.051	0.99	65.62
3	18.71	**0.006**	1.15	11.99	24.56	**0.003**	3.08	196.11
***Atypia***								
Low (< / = 2)	ref				ref			
High (> 2)	6.25	**0.001**	2.06	18.97	5.75	** < 0.001**	2.16	15.32
***Mitotic Index***								
< 5 mitoses/ 10HPF	ref				ref			
> 5 mitoses/ 10HPF	9.2	** < 0.001**	2.97	28.32	10.04	** < 0.001**	3.58	28.21
***LVI***								
No	ref				ref			
Yes	4.86	**0.041**	1.07	22.11	3.54	0.1	0.80	15.6
***Myxoid Component***								
No	ref				ref			
Yes	1.25	0.69	0.42	3.74	0.84	0.73	0.31	2.27
***Dedifferentiated Component***								
No	Ref				ref			
Yes	7.71	** < 0.001**	2.52	23.59	6.87	** < 0.001**	2.58	18.28

We constructed a multivariate analysis investigating the independent association of histopathologic factors with 5-year DSS and DFS (Table 5). First, we investigated the independent association of each *significant* “traditional” histopathologic marker with disease specific survival, which included atypia, mitotic activity and cellularity as described by Evans et al. [1] In a multivariate analysis controlling for potential confounding amongst these variables, neither high degree of atypia (HR 1.07, *p* = 0.96), high cellularity (HR 2.78, *p* = 0.29) or high mitotic activity (HR 3.83, *p* = 0.22) was significantly associated with DSS. Next, we investigated the independent association of “novel” variables with survival. When controlling for the above mentioned “traditional” histopathologic variables, presence of > 10% necrosis was significantly associated with disease specific survival (HR 3.58 [CI 1.09–11.71], *p* = 0.035). As 12 of 13 tumors classified as “high grade” contained dedifferentiation, we were unable to draw significant conclusions regarding the independent contribution of dedifferentiation relative to grade. Additionally, as only 3 patients presented with LVI, this variable could not be reliably included in a multivariate model.

**Table 5:** Multivariate analysis of individual “novel” histopathologic features and 5-year disease specific survival and disease free survival while controlling for traditional histopathologic variables

**Pathologic Factors**	**5-y DSS (HR)**	***p***	**95% Confidence Interval**	**5-y DFS** (**HR)**	***p***	**95% Confidence Interval**
***Necrosis***						
Low (< 10%)	ref			ref		
High (> 10%)	3.58	**0.035**	1.09–11.71	2.3	0.09	0.87–6.11
***Cellularity***						
Low (< / = 2)	ref			ref		
High (> 2)	3.98	0.9	0.65–24.31	2.54	0.30	0.43–14.84
***Atypia***						
Low (< / = 2)	ref			ref		
High (> 2)	1.16	0.9	0.12–11.29	0.9	0.93	0.09–9.2
***Mitotic Index***						
< 5 mitoses/ 10HPF	ref			ref		
> 5 mitoses/ 10HPF	2.48	0.43	0.26–23.78	4.98	0.15	0.57–43.64

When considering DFS (Table 5), we similarly investigated the independent association of each significant traditional histopathologic marker including atypia, mitotic activity and cellularity. In a multivariate analysis controlling for potential confounding variables, neither high degree of atypia (HR 0.81, *p* = 0.86), high cellularity (HR 2.23, *p* = 0.40) or high mitotic activity (HR 6.3, *p* = 0.09) was significantly associated with DFS. Next, we investigated the independent association of “novel” variables with DFS, utilizing grade as a composite marker for the above traditional variables. When controlling for grade, necrosis (HR 2.3, *p* = 0.09) was not significantly associated with disease free survival.

## Discussion

While the capacity for accurate diagnosis and successful treatment remains a challenge for patients with chondrosarcoma, perhaps of greater importance is the lack of prognostic factors that can reliably predict tumor behavior or patient survival for patients with this disease. To this end, the histopathologic features that are prognostic of survival have not been well established. The goal of this study was to identify histopathologic features of chondrosarcoma that are most associated with survival. Here we attempt to provide an evidenced based foundation for grading; the findings of this study suggest that traditional histopathologic features hold some prognostic value, but additional features, such as necrosis, may be important prognostic histopathologic factors worth considering at the time of grading.

While prognostic algorithms are well developed in other cancers, prognostic algorithms for chondrosarcoma are limited. An important component of traditional staging is grade. However, interobserver variability may limit prognostic value [16]. Thorkildsen et al. sought to propose a new risk stratification system utilizing both known and novel prognostic markers in chondrosarcoma, specifically questioning the utility of incorporating grade into prognostication at all [17]. They identified a patient profile that included tumor location, size and presence of a soft tissue component that, independent from grade, correlated closely with disease specific survival. Interestingly, when considering these variables as a composite, grade was no longer significantly associated with survival. In another study, Compton et al. criticized existing staging systems and attempted to provide an improved, evidence based tumor staging of chondrosarcoma [18]. In their “Vanderbilt Staging System,” they used the national cancer database (NCDB) to derive the prognostic value of various tumor and pathologic variables. This study had several notable findings. First, in a multivariate model, grade was significantly associated with survival; however, only grade 3 and dedifferentiated grades were associated with survival, while grade 2 showed no significant difference in survival as compared to grade 1. When comparing to the MSTS and AJCC staging systems, the Vanderbilt Staging System showed improved predictive accuracy. Clearly, the above studies show that traditional staging methods based on grade are incomplete and may lack prognostic utility. However, to improve upon existing nomograms and staging systems, what is needed are better individual markers of prognosis and standardization of grading. While both staging and grading speak to the aggressiveness of the tumor, they hold unique informative value. Staging provides a snapshot in time of the current behavior of the tumor at the gross level (size, extra osseus extension, metastasis); in contrast, grading attempts to evaluate the aggressiveness of the tumor at the cellular level, speaking to an attempt to predict biologic behavior even before those gross features become evident. While the two are intrinsically related, they are also necessarily distinct. For instance, when a tumor is already very large with a big soft tissue mass and distant spread- it stands to reason that the behavior is proving to be aggressive and the outcome poor. The fact that grading is less predictive just confirms that present histolopathologic methods are not able to predict the inherent biology of the tumor at a cellular level.

Our study suggests that traditional criteria for grading in chondrosarcoma may be missing important considerations, particularly necrosis and LVI. In univariate measures, the factors most associated with survival included high mitotic index, dedifferentiation, marked atypia, hypercellularity, presence of LVI and presence of necrosis. When controlling for confounding histopathologic variables, the factor that appeared to be most associated with survival was necrosis. Though included in the grading schema by Evans et al. [1], in our study, presence of myxoid change was not associated with OS or DFS. Although subject to debate, primary chondrosarcoma with prominent myxoid features is considered distinct from extraskeletal myxoid chondrosarcoma. Data to inform the prognostic significance of the presence of a myxoid component is limited; however, a study by Antonescu, et al. demonstrated that these tumors may have a more favorable course, particularly when compared to extraskeletal myxoid chondrosarcoma [19]. While traditional histological analysis of cartilage tumors would suggest that myxoid tissue is more likely to be associated with higher grade lesions, the present study could not discern an association with a survival benefit or detriment and the presence of myxoid tissue, suggesting the importance of this variable is less certain. Taken together, when considering grade and the biologic aggressiveness of a chondrosarcoma sample, a mitotic index of > 5 mits/ HPF appears to correlate with more aggressive tumor biology and tumor necrosis > 10% may similarly imply a more aggressive disease process; these factors should be considered carefully. Other metrics of grading (cellularity, atypia, LVI and myxoid component) hold value, but ultimately should be considered in the context of the above factors. Finally, grossing protocols might benefit from additional focus on the relationship between the tumor and surrounding soft tissues to potentially ensure appropriate identification of LVI.

When specifically considering the feature of dedifferentiation, all of the tumors in our cohort with a dedifferentiated component arose from a low-grade chondrosarcoma. Furthermore, most cases classified as “high grade” demonstrated the presence of dedifferentiation. This raises an interesting consideration as to how to classify dedifferentiated tumors. Many studies have suggested that these tumors should be classified separately from central conventional chondrosarcoma. However, the rationale for this is less clear, particularly as the dedifferentiated tumors lack a defining genetic feature [12, 20–22]. What is more, the existence of a lower grade component in all of our samples would suggest that dedifferentiated tumors should not be considered a separate subtype, but rather as progression of central conventional chondrosarcoma, as suggested by prior evidence [20, 22].

Necrosis is well known as a prognostic feature of many cancers. Within soft tissue sarcomas, tumor necrosis independent of neoadjuvant therapies has been associated with poorer overall survival. Carneiro et al. investigated the prognostic value of non-treatment derived necrosis in a subset of patients with soft tissue sarcoma and found that necrosis was independently associated with the development of metastatic disease [23]. For chondrosarcoma, the importance of necrosis is poorly understood. In the original consideration of a grading scheme for chondrosarcoma by Evans et al., necrosis was not considered as part of the histopathologic grading of these tumors [1]. Subsequently, some studies have attempted to quantify percent necrosis as a surrogate measure of chemotherapy effectiveness [24, 25]. For untreated chondrosarcomas though, there is limited evidence regarding the prognostic importance of tumor necrosis. The present findings suggest that tumor necrosis is an important factor to consider in the histopathologic grading of these tumors.

LVI represents a potentially novel feature for prognostic consideration in chondrosarcoma. LVI, characterized by tumor cell invasion into blood vessels and lymphatic spaces, is viewed to be one of the critical steps in tumor metastasis in many carcinomas [26, 27]. LVI has been closely correlated with poorer oncologic outcomes in several different carcinomas [26–29]. This has been particularly well borne out in breast cancer. A study by Rakha et al. validated this pathologic variable as prognostic of survival in cohort of 3812 patients with breast cancer and an assessment of LVI [26]. They found that, irrespective of grade, molecular class, and stage, LVI was significantly associated with survival. The prognostic role of LVI in bone and soft tissue sarcoma has only recently been elucidated. Recently, Ethun et al. investigated the national cancer database and identified 6169 patients with extremity soft tissue sarcoma with concurrent data on LVI [2]. They found that, even when controlling for potentially confounding variables, LVI was predictive of poorer overall survival.

Within bone sarcomas, though, this relationship remains less clear. Miao et al. investigated prognostic factors associated with dedifferentiated chondrosarcoma in a single institution study. In a subgroup analysis, they showed that LVI may be associated with local recurrence but not overall survival; however, these associations did not hold in multivariate methods. In the present study, in contrast to that of Miao et al., LVI was associated with worse 5-year overall survival in univariate methods but not with 5-year disease free survival. In a multivariate analysis including grade and other histopathologic variables, this relationship was no longer significant. Key differences exist between the two studies. Most notably, Miao et al. specifically investigated a cohort of patients with dedifferentiated tumors, while the present study incorporated tumors of all grades, an important factor that may account for a biologic reason for the differences seen. It should be noted that both studies are likely underpowered to show a significant difference in outcome based on LVI as only 9 patients in the Miao et al. and 3 patients in the present study had evidence of LVI on pathologic review. Taken in the context of the present study, it is possible that current grossing protocols are not providing appropriate deference to LVI. Perhaps this is simply a reflection of a need to better assess this histopathologic variable with improved grossing techniques and more systematic examination of the tumor periphery and surrounding soft tissue.

As this study is a retrospective review, it is important to acknowledge the limitations of this approach. Importantly, as a retrospective review, this study is subject to selection bias. Indeed, a large number of patients were excluded from analysis owing to lack of pathology available for review due to the fact that we are a referral center and original pathology did not originate at our institution. This may have affected the results of the study. However, the rigor of the exclusion criteria is also an inherent strength of the present body of work, as each patient underwent pathologic re-review by two pathologists. In a similar vein, grading of cartilaginous tumors is plagued by inherent poor inter-observer reliability of grading between pathologists. This is reflected by the fact that, upon re-review, several of specimens were subject to a change in grading. Again, though, the strength of this study is that all specimens were reviewed by the same two pathologists to decrease variability and reach consensus if necessary. It also warrants noting that, when assessing histopathologic variables, it is likely there is significant interplay amongst the variables. Inherently, tumors that are dedifferentiated have increasing cellularity, atypia and necrosis. As such, it may be difficult to differentiate the independent impact of these variables and provide a “ranked” importance, as we found in our study. Finally, as chondrosarcoma is a rare disease, we were limited by low patient numbers. Though we identified 51 patients, our study was underpowered to show a significant difference between histopathologic markers, particularly in a multivariate analysis. This likely explained why established risk factors, such as axial vs. appendicular location and larger tumor size, were not found to be statistically associated with prognosis. Additionally, while we did attempt to perform sub analyses looking at metastasis free survival and local recurrence free survival alone, we were underpowered to be able to identify significant differences, though we did note that the factors that are associated with increased risk of local recurrence and metastasis free survival are the same that are associated with DSS. Nonetheless, this study does call into question the existing grading schema for chondrosarcoma and suggests that additional histopathologic markers may warrant more routine consideration.

Within chondrosarcoma, the histopathologic features that are prognostic of survival have not been well established. This study provides an evidence-based means for considering histopathologic markers, such as tumor necrosis, mitotic index, cellularity and atypia, and their association with prognosis in chondrosarcoma. Our findings suggest that necrosis and LVI warrant further study, with a larger cohort, to evaluate the potential inclusion into the grading and/or prognostication of chondrosarcoma.

## Data Availability

The data presented in this study are available on request from the corresponding author. The data are not publicly available due to HIPAA and privacy constraints.
